# Editorial: Current status and recent advances in preclinical models for rare cancers

**DOI:** 10.3389/fonc.2025.1676020

**Published:** 2025-08-15

**Authors:** Ewa Krawczyk, Luciane R. Cavalli, Joanna Kitlinska

**Affiliations:** ^1^ Department of Pathology, Georgetown University Medical Center, Washington, DC, United States; ^2^ Instituto de Pesquisa Pele Pequeno Principe, Curitiba, Brazil; ^3^ Department of Oncology, Georgetown University Medical Center, Washington, DC, United States; ^4^ Department of Biochemistry and Molecular & Cellular Biology, Georgetown University Medical Center, Washington, DC, United States

**Keywords:** rare cancers, cancer models, preclinical disease models, translational cancer research, *in vitro* cancer models, *in vivo* cancer models

Despite advancements in diagnostic and successful therapies, cancer still poses an important threat to human health. Global burden of oncological diseases is significant – according to WHO, recently above 20 million new cases are diagnosed and more than 9 million people die per year because of various malignancies. Moreover, due to multiple factors, including aging of the societies, the number of cancer cases is predicted to increase in the near future. Therefore, all initiatives aimed at designing new diagnostic methods or novel therapeutic approaches are valuable and critically needed.

Preclinical assays, both *in vitro* and *in vivo*, remain a crucial part of cancer research. They are indispensable in every aspect of basic and translational medicine, from drug screening and repurposing, vaccine development, to researching the mechanisms of the disease. However, no preclinical model is perfect, and none of them recapitulates exactly the physiology and pathophysiology of a human organism. For example, cell culture models *in vitro* lack the complexity of heterogenic cancer structure and cellular composition, so using them to test interactions of cancer cells with microenvironment and immune system is challenging. As an alternative, three-dimensional preclinical models can be utilized for that purpose, although they are limited by a high cost and low-throughput. Conversely, animal models *in vivo* retain more relevance to humans, but they are not time and cost efficient, and ethical issues have been raised against them.

Nevertheless, preclinical disease models are important for cancer research, and there is an urgent need for the advancement in this area and development of new reliable methods. These new methods and techniques can facilitate research for commonly occurring cancers, but they are probably even more important for rare malignancies. Rare cancers research, due to their infrequent occurrence, limited availability of human tissues and scarce preclinical models, is especially challenging and can be often neglected. Therefore, an advancement in this area is essential.

This edition of Research Topic is a collection of research and review articles demonstrating the importance of various types of preclinical models in rare cancers research. Three of them focus on sarcomas, emphasizing the difficulties in diagnosis and insufficient treatment options for these diseases. Petrescu et al. discuss *in vitro* and *in vivo* models of bone sarcomas, Ewing sarcoma and osteosarcoma, ranging from two-dimensional cell cultures and three-dimensional organoids, microfluidic platforms and xenograft models to genetically engineered mouse, zebrafish and *Drosophila* models. The authors point out the need for advancement of complex systems, recapitulating cancer microenvironment and the disease stage more precisely. Sankhe et al. focus on rhabdomyosarcoma fusion oncogenes, and describe available models to study them. They state that the establishment of new reliable models is needed to understand the biology and function of rhabdomyosarcoma oncogenes and their role in tumorigenesis. Li and Piesner report the generation of both *in vitro* and *in vivo* models to investigate metastasis in clear cell sarcoma of soft tissue (CCSST), a rare and aggressive tumor driven by EWSR1-ATF1 or EWSR1-CREB fusion proteins. Their analysis revealed that not all the fusion variants are constitutively active and that among four patient-derived cell lines tested, only one of them, CCS292, demonstrated invasive and migratory properties, and when injected into mice developed metastases in multiple organs, confirming its use as a robust model for studying CCSST dissemination.

The second research study in the Research Topic by McLean et al. reports a porcine model of Neurofibromatosis Type 1 (NF1) that overcomes key limitations of previous mouse models. The authors analyzed spontaneous neurofibromas in NF1 pigs using single-cell RNA sequencing, revealing a heterogeneous tumor microenvironment marked by M2 macrophage polarization, immunosuppressive signaling, extracellular matrix remodeling, and nerve regeneration pathways. These results confirm that the porcine model closely resembles human neurofibromas and offers a powerful platform for studying NF1 tumor biology and testing immune-based therapies. Similarly, Lemm et al. in their methods article demonstrate generation of two genetically modified *in vitro* models of pheochromocytoma varying in their resistance to radiation therapy. These radioresistant cell lines were subsequently used to establish 3D *in vitro* and *in vivo* models that can be crucial for understanding the metastatic processes that occur after the radionuclide therapy of the disease.

Three remaining Reviews summarize the current status and the advances in preclinical models for other rare cancers. Hatanaka and Braunig explore the existing preclinical models of supratentorial ependymomas, describe their strengths and limitations, as well as potential clinical applications. Various models for pediatric low-grade gliomas, their genetic alterations, strengths and weaknesses, and potential implications for the patients are demonstrated by Yvone and Braunig. Additionally, Kes et al. extensively discuss the current status of 2D and 3D cell culture systems implemented to study several aspects of cancer cell metabolism in various rare and common cancer models *in vitro*.

Altogether, excellent research and review articles collected in this Research Topic clearly demonstrate the current status as well as exciting advancements in rare cancers modeling, that may greatly facilitate and accelerate the development of new diagnostic and therapeutic approaches for these challenging malignancies.

