# Correction: Editorial: Current status and recent advances in preclinical models for rare cancers

**DOI:** 10.3389/fonc.2025.1697286

**Published:** 2025-09-23

**Authors:** Ewa Krawczyk, Luciane R. Cavalli, Joanna Kitlinska

**Affiliations:** ^1^ Department of Pathology, Georgetown University Medical Center, Washington, DC, United States; ^2^ Instituto de Pesquisa Pele Pequeno Principe, Curitiba, Brazil; ^3^ Department of Oncology, Georgetown University Medical Center, Washington, DC, United States; ^4^ Department of Biochemistry and Molecular & Cellular Biology, Georgetown University Medical Center, Washington, DC, United States

**Keywords:** rare cancers, cancer models, preclinical disease models, translational cancer research, *in vitro* cancer models, *in vivo* cancer models

Affiliation “Department of Oncology, Georgetown University Medical Center, Washington, DC, United States” was omitted for author Luciane R. Cavalli. This affiliation has now been added for author Luciane R. Cavalli.

Affiliation “Department of Biochemistry and Molecular & Cellular Biology, Georgetown University Medical Center, Washington, DC, United States” was omitted for author Joanna Kitlinska. Erroneously given as “Department of Pathology, Georgetown University Medical Center, Washington, DC, United States.

The original version of this article has been updated.

